# Mild Hypertransaminasemia in Primary Care

**DOI:** 10.1155/2013/256426

**Published:** 2013-04-10

**Authors:** Said A. Al-Busafi, Nir Hilzenrat

**Affiliations:** ^1^Department of Medicine, College of Medicine and Health Science, Sultan Qaboos University, P.O. Box 35, 123 Muscat, Oman; ^2^Department of Gastroenterology, Jewish General Hospital, McGill University, Montreal, QC, Canada H3T 1E2

## Abstract

The liver enzymes, alanine transaminase (ALT) or aspartate transaminase (AST), are commonly used in clinical practice as screening as well as diagnostic tests for liver diseases. ALT is more specific for liver injury than AST and has been shown to be a good predictor of liver related and all-cause mortality. Asymptomatic mild hypertransaminasemia (i.e., less than five times normal) is a common finding in primary care and this could be attributed to serious underlying condition or has transient and benign cause. Unfortunately, there are no good literatures available on the cost-effectiveness of evaluating patients with asymptomatic mild hypertransaminasemia. However, if the history and physical examination do not suggest a clear cause, a stepwise approach should be initiated based on pretest probability of the underlying liver disease. Nonalcoholic fatty liver disease is becoming the most common cause of mild hypertransaminasemia worldwide. Other causes include alcohol abuse, medications, and hepatitis B and C. Less common causes include hemochromatosis, *α*1-antitrypsin deficiency, autoimmune hepatitis, and Wilson's disease. Nonhepatic causes such as celiac disease, thyroid, and muscle disorders should be considered in the differential diagnosis. Referral to a specialist and a possible liver biopsy should be considered if persistent hypertransaminasemia for six months or more of unclear etiology.

## 1. Introduction

The term “liver function tests (LFT)” is commonly used in clinical practice when referring to liver enzymes. However this term is misleading since these tests primarily assess liver injury rather than liver function. In addition, this term also implies that these tests are solely of hepatic origin, but in fact, they are not always specific for the liver. The serum aminotransaminases, alanine transaminase (ALT, formally serum glutamic-pyruvic transaminase (SGPT)) and aspartate transaminase (AST, formally serum glutamic-oxaloacetic transaminase (SGOT)), are frequently obtained in primary care for screening and diagnosis of liver diseases and have therefore led to increased number of asymptomatic patients with mild hypertransaminasemia (i.e., less than five times the upper limit of normal) [1]. A population-based survey in the United States found that hypertransaminasemia was present in 8.9 percent of the population [2]. Although the majority of these individuals will have benign conditions, a subgroup will have underlying significant liver diseases that require further evaluation and therapeutic intervention.

An audit of primary care practices found that these abnormalities are often not adequately investigated, missing an important chance of identifying treatable chronic liver disease [3]. Given the importance and frequency of this issue, the primary care physician should develop a rational approach to the management of mild hypertransaminasemia. There are several published guidelines for the evaluation of asymptomatic hypertransaminasemia [1, 4, 5], which are mainly based on expert opinions, and a very limited number of clinical studies. Understanding the basic disease processes that cause mildly hypertransaminasemia and the epidemiology of each disease can help guide the clinical evaluation and efficient use of laboratory testing.

## 2. Literature Review

We searched PubMed using the following keywords: “elevated,” “liver tests,” “liver enzymes,” “transaminase,” and “aminotransferase.” The articles were excluded from our paper if they did not pertain to the topic of hypertransaminasemia, or if they were in a language other than English. The rest of our recommendations are based on data from few retrospective studies, case series (Level B), or expert consensus guidelines (Level C).

## 3. Transaminases as Markers of Liver Disease

The serums ALT and AST are the most reliable and sensitive indicators of hepatocellular injury. Their levels can be elevated in a variety of hepatic disorders. ALT, which is present mainly in the cytosol of liver and much less in the other tissues, is the most specific for liver disease [6], whereas AST, which has cytosolic and mitochondrial forms, is less specific for liver disease as it is found, in addition to the liver, in the heart, skeletal muscle, kidneys, brain, pancreas, lungs, leukocytes, and erythrocytes. Although levels of ALT and AST can be extremely elevated (greater than 15 times the upper limit of normal (ULN) in cases of acute liver injury related to drugs, toxins, ischemia, and hepatitis), elevations less than 5 times the ULN are much more common in primary care practice.

Although one study suggested that the majority of asymptomatic individuals (88%) with mild ALT elevations do not have identifiable causes [7], a Scandinavian study of 151 consecutive patients with mild to moderate elevations (42–300 IU/L) of ALT levels for at least 6 months revealed that identifiable causes of liver disease were more common [8]. Diagnoses included NAFLD in 42%, chronic HCV in 15.3%, presumed alcoholic liver disease in 8%; autoimmune hepatitis, primary biliary cirrhosis, and alpha1 antitrypsin deficiency were much less common. Both of these studies were conducted in the era of less sensitive HCV testing and therefore the true prevalence of HCV infection could not be ascertained.

The AST/ALT ratio can suggest certain disease patterns. In most of the liver diseases (acute or chronic), the ratio is less than or equal to 1 such as nonalcoholic fatty liver disease (NAFLD) and viral hepatitis (B or C) [9], whereas, an AST/ALT ratio greater than 2 characteristically is present in alcoholic hepatitis [10] and a ratio greater than 4 suggests Wilson's disease [11]. In addition, smaller increases in the ratio to values greater than 1.0 suggest the presence of cirrhosis in different causes of nonalcoholic liver diseases such as NAFLD, hepatitis B, and hepatitis C [9]. Therefore, a ratio greater than 1.0 in a cirrhotic patient is not necessarily indicative of alcoholic liver disease. Furthermore, while these ratios are suggestive of certain conditions, there is too much overlap between groups to rely on them exclusively when making a diagnosis.

In addition, aminotransferase levels may be normal in a subset of liver disease despite advanced histologic features. In other words there is a lack of correlation between the level of transaminases and severity of the histologic damage in patients with chronic hepatitis such as nonalcoholic steatohepatitis [12] and hepatitis C [13, 14].

## 4. ALT: What Is Normal Value?

ALT is commonly used in clinical practice as a screening, diagnostic, and monitoring test for liver diseases [15]. However, accepted definitions and uniform measurements of the normal range have been an unsettled issue. Therefore, ALT level, which differentiates between asymptomatic persons who have liver disease and those who do not, is still uncertain. Several studies have addressed the ULN of ALT. Two recent reports from United States showed a wide variation in ALT ULN values across laboratories [16, 17]. Some of this variation may be attributed to the use of different instruments or methods to perform the measurement or to differences in reference populations used to define the ULN [18].

The reference ranges for routine laboratory tests are determined based on 2 methods, either on values obtained from healthy individuals or on health outcomes [19]. The former, which is the commonly used method, involves selecting apparently healthy individuals and setting the reference limits (upper and lower ends of the “normal range”) to include the central 95% of values for the test, whereas, the use of health outcome-based reference ranges requires that there is a high degree of standardization across different labs, and a precise relationship exists between adverse health outcome and a discrete level of the lab value.

When using the central 95% of ALT values of the “normal” population, 5% will have abnormal ALT (2.5% are above and 2.5% are below the normal range) which means that 2.5% of normal individual will have abnormally high results. In addition, the current ULN for ALT (40 IU/L in men, 30 IU/L in women) has been established in 1980s when it was introduced as a surrogate marker for screening of hepatitis C among blood donors and before the implementation of anti-HCV testing and restrictive behavioral criteria for donor selection. This explains why ALT ULN is different between labs as those individuals were only “apparently healthy,” and have other reasons for increased ALT. The most important of these are Hepatitis C and NAFLD. Other factors also known to cause increased ALT include medications, herbal supplements, and excessive ethanol ingestion [19]. In a study of blood donors conducted in Milan, Italy, which included individuals at lowest risk of liver disease (no hepatitis B or C, not overweight, alcohol abuse or taking any medications, and those with normal levels of cholesterol, triglycerides, or glucose), the ULN was 30 IU/L in men and 19 IU/L in women [20]. In addition, a similar result for men was obtained in a South Korean outcome study of more than 90,000 men followed for liver related mortality over 8 years, where cut-off value of 30 IU/L was the best predictive of liver disease in men [21]. This new ULN was shown to increase sensitivity for detection of hepatitis C virus among blood donors from 40% to 61% with very slight decline in specificity from 97.6% to 95.5% [20]. Using the old ULN of ALT, 50% of the individuals with increased ALT had no obvious cause for liver injury, but most of them (85%) had evidence of steatosis on liver ultrasound. Therefore, it is clear that the current different ULN used by different labs is insensitive to the presence of liver disease and does not detect limits at which risk of liver mortality seems to increase. In addition, this wide variation in levels considered “abnormal” between different labs leads to differences in the recognition of liver diseases and decision to treat hepatitis B virus [22]. In the other hand, using the new lower ULN might lead to labeling a large number of the populations as having liver injury when the cost of evaluation would be high and its utility is still uncertain.

Several strategies should be used in order to establish the “healthy” range of ALT of the target population including using similar lab methods and excluding individuals with risk factors for liver diseases. We, the authors of this review, commonly use 30 IU/L for men and 19 IU/L for women as a reference for ULN of ALT when dealing with patients at high risk for liver diseases.

## 5. ALT as Predictor of Health Based Outcomes

While ALT is useful as an initial test in detecting liver disease, emerging data highlight its potential value as a measure of overall health and survival. There are several observational studies which have shown a strong relationship between ALT activity and liver and all-cause mortality. The strongest is a population-based study from Korea which included a cohort of 142,055 participants of ages between 35 and 59 years followed up to 10 years, when death certificates were used to determine survival and causes of death [21]. In this study, the risk of death from liver disease started to increase at ALT value above 20 IU/L. Moreover, increased ALT has been associated with the risk of death from all-cause or cardiovascular disease with risk begining to increase even at level below current ULN [21, 23, 24]. While mortality risk may be due to unknown liver disease, it may partly be explained by the presence of metabolic syndrome in patients with NAFLD, in addition to alcohol consumption, which are linked to nonliver health risks.

## 6. Initial Approach to the Evaluation of Mild Hypertransaminasemia

**Figure 1:**
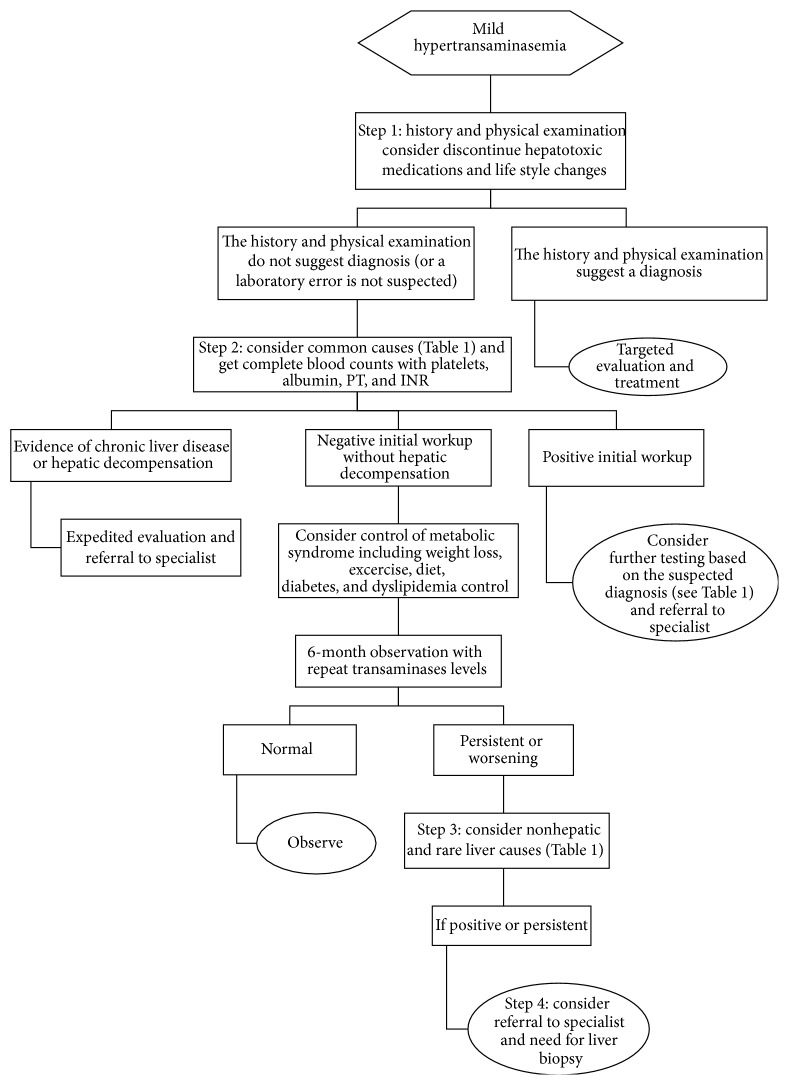
Management algorithm of mild hypertransaminasemia [1, 4, 5].

The clinical significance of mild hypertransaminasemia must be interpreted in the context of the clinical presentation. In general, symptomatic patients (i.e. with signs or symptoms of chronic liver disease or evidence of hepatic decompensation e.g. ascites, encephalopathy, coagulopathy, or portal hypertension) should be evaluated and treated in a more expeditious manner than asymptomatic patients with normal physical exam. Unfortunately, there are no data available on the cost-effectiveness of evaluating patients with asymptomatic mild hypertransaminasemia, nor on the natural history of the potential liver disease in these patients. However, given the high prevalence of this abnormality in the primary care settings and the significant costs of an extensive evaluation, rational stepwise approach should be guided by the pretest probability of the underlying liver disease, the pattern of abnormalities, and suggestive features obtained from the history and physical examination. The following sections will provide this approach based on the published guidelines [1, 4, 5] (Figure 1).

### 6.1. History, Physical Examination, and Life Style Changes

A detailed history and physical examination are essential for the initial evaluation to determine whether the liver injury is acute or chronic (defined as ≥6 months), the underlying cause, and associated comorbidities. Important considerations include:

- the presence of any accompanying symptoms such as abdominal pain, fever, and weight loss or symptoms of liver dysfunction such as jaundice, confusion, ascites, and leg swelling;
- the exposure to any medication including prescription and over-the-counter medications as well as herbal therapies;
- occupational exposure to other hepatotoxins and alcohol consumption;
- risk factors for viral hepatitis including possible parenteral exposures including transfusions, intravenous and intranasal drug use, tattoos, and sexual activity;
- family history of liver disease or other autoimmune disorders.

The physical examination should include assessment for signs of metabolic syndrome, a risk factor for NAFLD, such as hypertension, obesity (body mass index, BMI, and waist circumference), dyslipidemia (corneal urcus and xanthomas), and insulin resistance (acanthosis nigricans). In addition, patients should be examined for findings suggesting the presence of liver disease, including the assessment for stigmata of chronic liver disease (e.g., spider nevi, palmar erythema, gynecomastia, etc.), splenomegaly (suggestive of portal hypertension), and ascites.

If the history and physical examination suggest a particular diagnosis, a targeted testing should follow. On the other hand, more than 30% of individuals with initially mild hypertransaminasemia will have normal levels on retesting [25]. Therefore, if ALT elevation is less than 2 times normal and if the history and physical examination do not suggest an etiology or a laboratory error is strongly suspected, it is reasonable to repeat the test in four weeks [1, 4]. However, it should be cautioned that hepatitis C may present with fluctuating elevated liver enzymes levels.

In addition, a period of effective lifestyle changes can be initiated including complete abstinence from alcohol, control of diabetes and hyperlipidemia, weight loss in overweight patients, and stopping or changing potentially hepatotoxic medications and supplements (Figure 1). Such lifestyle changes directly impact several of the causes of mild hypertransaminasemia and may be all that is needed to correct the abnormalities [11] (Table 1).

### 6.2. Look for Common Causes

Additional laboratory tests for common causes of liver injury should be obtained when history and physical examination show no obvious cause (Table 1). Hepatitis B, hepatitis C, and hemochromatosis should be considered [4]. These conditions were found to be responsible for mild hypertransaminasemia in only 31% of patients [26]. The majority of the patients (69%) had unexplained elevations, but there were strong associations with markers of metabolic syndrome and therefore may represent NAFLD [26]. This observation was also confirmed in a previous study which showed that NAFLD was responsible of at least 80% of asymptomatic hypertransaminasemia after other causes were ruled out [27]. The measurement of a complete blood count with platelet count, coagulation profile, and albumin should be considered if liver dysfunction is suspected.

### 6.3. Consider Nonhepatic Causes and Rare Liver Conditions

If the etiology was not reached despite the above workups, observation with lifestyle changes should be undertaken for up to 6 months [4]. If hypertransaminasemia persists or worsens, the patient should be reevaluated and a further diagnostic testing should be performed, if necessary. The next step should include testing for nonhepatic causes, including muscles and thyroid diseases, as well as rare causes like celiac disease, based on the clinical scenario (Table 1). In addition, rare liver diseases, including Wilson's disease, Alpha-1 antitrypsin deficiency, and autoimmune hepatitis, should be considered at this stage (Table 1).

### 6.4. Referral to a Specialist

Consultation with a gastroenterologist or hepatologist should be considered for the following groups of patients [5]:

- patients with unexplained hypertransaminasemia on two occasions, a minimum of 6 months apart despite life style changes;
- symptoms or signs of liver decompensation (stigmata of chronic liver diseases, ascites, and hepatic encephalopathy);
- evidence of liver dysfunction (high bilirubin, low albumin, and prolonged PT or INR);
- evidence of liver disease where treatment beyond the withdrawal of implicated agent (alcohol or drugs) is warranted, for example, hepatitis B, hepatitis C, autoimmune hepatitis, Wilson's disease, hemochromatosis, and NAFLD.

## 7. Indications for Liver Biopsy

In general, liver biopsy has three major roles: to make a firm diagnosis (or exclude a diagnosis of any serious or significant liver disease), for assessment of prognosis (disease staging), and/or to assist in making therapeutic management decisions. In cases of mild hypertransaminasemia, it is often considered in patients in whom all noninvasive tests were negative or in patients in whom a specific liver disease has been considered but has not yet been confirmed, for example, Wilson's disease and Alpha-1 antitrypsin deficiency. While it remains less likely that the biopsy will provide a diagnosis or lead to changes in management, it is often reassuring for the patient and clinician to know that there is no serious disorder.

## 8. Conclusion 

Mild hypertransaminasemia is a common finding in primary care practice. Unfortunately, there are no data available on the cost-effectiveness of evaluating such patients. If the history and physical examination do not suggest a cause, a stepwise approach should be initiated based on the pretest probability of the underlying liver disease. Patients with an abnormal albumin or prothrombin time or with evidence of chronic liver disease and/or hepatic decompensation should typically have more expeditious evaluations preferably by a specialist. Referral to a specialist is also recommended if unexplained asymptomatic hypertransaminasemia remains elevated for six months or more.

## Figures and Tables

**Table 1:** Causes of mild hypertransaminasemia, clinical clues and initial diagnostic testing [1, 4, 5].

Etiology	Clinical clues	Initial diagnostic testing
Common causes		

Drugs (including prescribed, over the counter, illicit drug use and herbals)	(i) Lack of illness prior to taking the drug (ii) Clinical illness or biochemical abnormalities developing after beginning the drug (iii) Improvement after the drug is withdrawn	History
Alcohol abuse	Excessive alcohol consumption, AST/ALT ratio ≥ 2.0	Accurate history, CAGE questionnaire, AST/ALT ratio, *γ*-glutamyl transpeptidase level (GGT)
Nonalcoholic fatty liver disease (NAFLD)	Evidence of metabolic syndrome (dyslipidemia, hypertension, diabetes or central obesity) AST/ALT ratio < 1.0	Fasting lipid profile, glucose level; consider ultrasonography to detect hepatic steatosis
Hepatitis B	High risk factors including (Immigration from endemic countries, high risk sexual behavior, and intravenous drug use)	Hepatitis B surface antigen (HBsAg), Hepatitis B surface antibody (HBsAb), Hepatitis B core antibody (HBcAb)
Hepatitis C	Parenteral exposure (blood transfusions, intravenous drug use, occupational), tattoos, body piercing, and high risk sexual behavior	Hepatitis C virus antibody testing
Hereditary Hemochromatosis	Family history	Transferrin saturation and ferritin levels

Less common causes		

Autoimmune hepatitis	Personal of family history of other autoimmune diseases	Immunoglobulin G levels, Serum protein electrophoresis (SPEP); antinuclear antibodies (ANA), smooth muscle antibodies (SMA) and liver-kidney microsomal antibodies testing (LKMA)
Wilson's disease	Younger than 40 years, neuropsychiatric symptoms, Kayser-Fleischer rings	Serum ceruloplasmin level and ophthalmologist consultation to rule out Kayser-Fleischer rings
*α*1-antitrypsin deficiency	Early-onset emphysema, family history	Serum *α*1-antitrypsin level and SPEP

Non-Hepatic causes		

Muscle disorders	Muscle weakness and pain, strenuous exercise	Creatine kinase (CK) and aldolase levels
Thyroid disorders	Signs and symptoms of hypo- or hyperthyroidism	Thyroid-stimulating hormone (TSH) level
Celiac disease	Diarrhea, abdominal pain, malabsorption	Tissue transglutaminase antibody (TTG) testing

## References

[1] Pratt D. S., Kaplan M. M. (2000). Evaluation of abnormal liver-enzyme results in asymptomatic patients. *The New England Journal of Medicine*.

[2] Ioannou G. N., Boyko E. J., Lee S. P. (2006). The prevalence and predictors of elevated serum aminotransferase activity in the United States in 1999–2002. *American Journal of Gastroenterology*.

[3] Sherwood P., Lyburn I., Brown S., Ryder S. (2001). How are abnormal results for liver function tests dealt with in primary care? Audit of yield and impact. *British Medical Journal*.

[4] Green R. M., Flamm S. (2002). AGA technical review on the evaluation of liver chemistry tests. *Gastroenterology*.

[5] Minuk G. Y. (1998). Canadian association of gastroenterology practice guidelines: evaluation of abnormal liver enzyme tests. *Canadian Journal of Gastroenterology*.

[6] Rej R. (1978). Aspartate aminotransferase activity and isoenzyme proportions in human liver tissues. *Clinical Chemistry*.

[7] Kundrotas L. W., Clement D. J. (1993). Serum alanine aminotransferase (ALT) elevation in asymptomatic US Air Force basic trainee blood donors. *Digestive Diseases and Sciences*.

[8] Mathiesen U. L., Franzén L. E., Frydén A., Foberg U., Bodemar G. (1999). The clinical significance of slightly to moderately increased liver transaminase values in asymptomatic patients. *Scandinavian Journal of Gastroenterology*.

[9] Williams A. L. B., Hoofnagle J. H. (1988). Ratio of serum aspartate to alanine aminotransferase in chronic hepatitis. Relationship to cirrhosis. *Gastroenterology*.

[10] Cohen J. A., Kaplan M. M. (1979). The SGOT/SGPT ratio. An indicator of alcoholic liver disease. *Digestive Diseases and Sciences*.

[11] Krier M., Ahmed A. (2009). The asymptomatic outpatient with abnormal liver function tests. *Clinics in Liver Disease*.

[12] Mofrad P., Contos M. J., Haque M. (2003). Clinical and histologic spectrum of nonalcoholic fatty liver disease associated with normal ALT values. *Hepatology*.

[13] Shakil A. O., Conry-Cantilena C., Alter H. J. (1995). Volunteer blood donors with antibody to hepatitis C virus: clinical, biochemical, virologic, and histologic features. *Annals of Internal Medicine*.

[14] Haber M. M., West A. B., Haber A. D., Reuben A. (1995). Relationship of aminotransferases to liver histological status in chronic hepatitis C. *American Journal of Gastroenterology*.

[15] Kim W. R., Flamm S. L., di Bisceglie A. M., Bodenheimer H. C. (2008). Serum activity of alanine aminotransferase (ALT) as an indicator of health and disease. *Hepatology*.

[16] Dutta A., Saha C., Johnson C. S., Chalasani N. (2009). Variability in the upper limit of normal for serum alanine aminotransferase levels: a statewide study. *Hepatology*.

[17] Neuschwander-Tetri B. A., Ünalp A., Creer M. H. (2008). Influence of local reference populations on upper limits of normal for serum alanine aminotransferase levels. *Archives of Internal Medicine*.

[18] Ruhl C. E., Everhart J. E. (2012). Upper limits of normal for alanine aminotransferase activity in the United States population. *Hepatology*.

[19] Dufour D. R., Lott J. A., Nolte F. S., Gretch D. R., Koff R. S., Seeff L. B. (2000). Diagnosis and monitoring of hepatic injury. I. Performance characteristics of laboratory tests. *Clinical Chemistry*.

[20] Prati D., Taioli E., Zanella A. (2002). Updated definitions of healthy ranges for serum alanine aminotransferase levels. *Annals of Internal Medicine*.

[21] Kim H. C., Nam C. M., Jee S. H., Han K. H., Oh D. K., Suh I. (2004). Normal serum aminotransferase concentration and risk of mortality from liver diseases: prospective cohort study. *British Medical Journal*.

[22] Lok A. S. F., McMahon B. J. (2007). Chronic hepatitis B. *Hepatology*.

[23] Tae H. L., Kim W. R., Benson J. T., Therneau T. M., Melton L. J. (2008). Serum aminotransferase activity and mortality risk in a United States community. *Hepatology*.

[24] Goessling W., Massaro J. M., Vasan R. S., D'Agostino R. B., Ellison R. C., Fox C. S. (2008). Aminotransferase levels and 20-year risk of metabolic syndrome, diabetes, and cardiovascular disease. *Gastroenterology*.

[25] Lazo M., Selvin E., Clark J. M. (2008). Brief communication: clinical implications of short-term variability in liver function test results. *Annals of Internal Medicine*.

[26] Clark J. M., Brancati F. L., Diehl A. M. (2003). The prevalence and etiology of elevated aminotransferase levels in the United States. *American Journal of Gastroenterology*.

[27] Daniel S., Ben-Menachem T., Vasudevan G., Ma C. K., Blumenkehl M. (1999). Prospective evaluation of unexplained chronic liver transaminase abnormalities in asymptomatic and symptomatic patients. *American Journal of Gastroenterology*.

